# The influence of sulfur and hair growth on stable isotope diet estimates for grizzly bears

**DOI:** 10.1371/journal.pone.0172194

**Published:** 2017-03-01

**Authors:** Garth Mowat, P. Jeff Curtis, Diana J. R. Lafferty

**Affiliations:** 1 Natural Resource Science Section, BC Ministry of Forests, Lands and Natural Resource Operations, Nelson, British Columbia, Canada; 2 Department of Earth and Environmental Sciences, The University of British Columbia Okanagan Campus, Kelowna, British Columbia, Canada; 3 Department of Forestry and Environmental Resources, Program in Wildlife, Fisheries and Conservation Biology, North Carolina State University, Raleigh, North Carolina, United States of America; Institute of Zoology, CHINA

## Abstract

Stable isotope ratios of grizzly bear (*Ursus arctos*) guard hair collected from bears on the lower Stikine River, British Columbia (BC) were analyzed to: 1) test whether measuring δ^34^S values improved the precision of the salmon (*Oncorhynchus* spp.) diet fraction estimate relative to δ^15^N as is conventionally done, 2) investigate whether measuring δ^34^S values improves the separation of diet contributions of moose (*Alces alces*), marmot (*Marmota caligata*), and mountain goat (*Oreamnos americanus*) and, 3) examine the relationship between collection date and length of hair and stable isotope values. Variation in isotope signatures among hair samples from the same bear and year were not trivial. The addition of δ^34^S values to mixing models used to estimate diet fractions generated small improvement in the precision of salmon and terrestrial prey diet fractions. Although the δ^34^S value for salmon is precise and appears general among species and areas, sulfur ratios were strongly correlated with nitrogen ratios and therefore added little new information to the mixing model regarding the consumption of salmon. Mean δ^34^S values for the three terrestrial herbivores of interest were similar and imprecise, so these data also added little new information to the mixing model. The addition of sulfur data did confirm that at least some bears in this system ate marmots during summer and fall. We show that there are bears with short hair that assimilate >20% salmon in their diet and bears with longer hair that eat no salmon living within a few kilometers of one another in a coastal ecosystem. Grizzly bears are thought to re-grow hair between June and October however our analysis of sectioned hair suggested at least some hairs begin growing in July or August, not June and, that hair of wild bears may grow faster than observed in captive bears. Our hair samples may have been from the year of sampling or the previous year because samples were collected in summer when bears were growing new hair. The salmon diet fraction increased with later hair collection dates, as expected if samples were from the year of sampling because salmon began to arrive in mid-summer. Bears that ate salmon had shorter hair and δ^15^N and δ^34^S values declined with hair length, also suggesting some hair samples were grown the year of sampling. To be sure to capture an entire hair growth period, samples must be collected in late fall. Early spring samples are also likely to be from the previous year but the date when hair begins to grow appears to vary. Choosing the longest hair available should increase the chance the hair was grown during the previous year and, maximize the period for which diet is measured.

## Introduction

Grizzly bears living in coastal North America have several possible life history strategies that incorporate various amounts salmon in their diet. One possible life history strategy is for grizzly bears to forage on salmon whenever they are available. This strategy brings bears into close contact with each other because places where bears can efficiently catch spawning salmon are often few and clumped, which may increase the risk of injury for smaller bears [1]. Further, bears that forage predominantly on salmon are larger, have greater reproductive output, and often occur at higher densities than other grizzly bear populations [2].

A second foraging strategy for bears living in coastal North America is to forage predominantly on the abundant vegetation available in this wet ecosystem and consume other protein sources opportunistically. When not foraging on salmon, grizzly bears tend to focus foraging efforts on emergent and easily digestible vegetation, the starchy roots of some plants and, fruits of many shrub species [3]. All three forms of vegetation are available in large quantities in the Stikine River valley because the treeline is very low, the valley bottoms are flat, moist and regularly disturbed and, there are large numbers of snow avalanche chutes that are maintained in early seral forb or shrub dominated meadows.

A third possible foraging strategy is to preferentially hunt the various mammals available. For example, grizzly bears are known to prey on moose and caribou (*Rangifer tarandus*) in the spring, particularly calves, which are most vulnerable during the first 6–8 weeks of life [4,5]. Moose were not abundant in this ecosystem [6] but they are a potential source of protein for bears, while caribou were absent. But grizzly bears in the Stikine River area may have somewhat unique food habits. Anecdotal reports suggest that some grizzly bears regularly hunt mountain goats or, following the spring thaw, that some bears scavenge the remains of mountain goats killed in avalanches and preserved in snow during the winter months. Marmots appear abundant and may also be an important food source for grizzlies during the summer and fall. Throughout much of their North American range, neither mountain goats nor marmots are abundant and are not known to be important food sources for grizzly bears [7,8]. However, MacHutchon and Mahon [9] suggested that marmots were an important food for grizzly bears during the fall in the Babine River watershed of central BC. Understanding the importance of these species in the diet of local bear populations would help to understand the importance of habitats used by marmots and goats to bears because both species occur in specific habitats.

All three foraging strategies may be combined within and among years, especially by female bears. By employing diverse foraging strategies female bears may minimize predation risk of their cubs [3]. Also, flexible foraging behavior gives bears the opportunity to switch foods when a preferred food is less abundant or unavailable. Understanding the different foraging strategies employed by individual bears could aid in predicting development impacts, planning mitigation, and designing monitoring strategies.

Salmon are known to be an important food source for bears in many coastal areas and this diet component cannot be accurately measured using traditional scat-based diet analyses because fish are mostly protein and little fish remains are found in scats [10] and, scats from pure protein meals are not preserved for long in the environment. Stable isotope analysis permits the estimation of the proportional contribution of major food items to the assimilated diet of a consumer. When bears are sampled using hair traps and individually identified via genetic analysis, hair can be used to index individual bear diet during the period of hair growth [11,12]. In addition, seasonal diet can be calculated by sectioning hairs by length [13,14]. Hair growth was observed from late spring to early fall in captive bears [15] but hair growth has not been documented in wild bears. Grizzly bears appear to have a full covering of guard hair, at least on their back, throughout the summer (B. McLellan, BC Ministry of FLNRO, personal communication) which suggests some variation in the beginning of re-growth of guard hairs. Stable carbon and nitrogen isotope values have been used to assign diet for bears in many recent studies however, assigning more than 3 food types with 2 markers is often imprecise because some food items have similar isotopes signatures [16]. Adding a third marker such as sulfur may allow greater resolution of food types and more precise assignment of the salmon fraction [15], and perhaps other foods.

Sulfur is an abundant element in the ecosphere and in organic tissue. It is also important to protein structure because sulfur is a necessary element in two amino acids [17]. Sulfur isotope ratios are highly variable in rock and soil but nearly fixed in the ocean environment [17]. This variability makes it difficult to create general baseline signatures for diet analysis unless the diet item is derived from the ocean. Sulfur and nitrogen isotope values are commonly related, presumably because both index the protein intake and growth in the consumer. However there is little fractionation of sulfur during somatic growth hence sulfur does not indicate trophic position as effectively as nitrogen. Nehlich [17] provides a very thorough recent review of the geochemistry of sulfur and its applications in diet and place of origin analysis.

The objectives of this study were to: 1) examine whether adding δ^34^S values to a mixing model that included δ^15^N and δ^13^C more precisely estimated salmon in the diet of grizzly bears compared to δ^15^N and δ^13^C alone, 2) assess whether δ^34^S values, in conjunction with δ^13^C and δ^15^N, would improve the proportional contributions of moose, marmot, and mountain goat to the diet of grizzly bears and, 3) examine the sectioning of hair by length to index seasonal trends in the above diet measures, and 4) consider the period of hair growth using hair length and sample collection date compared to the arrival date of salmon in this system.

### Study area

Our Stikine River study area was located in north-western British Columbia approximately 1000 km north of Vancouver, adjacent to the Alaska panhandle, and approximately 90 km northeast of Wrangell, Alaska. The area is transitional between coastal and interior climatic influences. Low elevation forests are conifer dominated while alpine tundra dominated above about 1000 m. Topography is very rugged and includes many large glaciers and steep, rocky peaks above 2,000 m. A diversity of habitat types exist within the study area including extensive floodplain habitat and wetlands, as well as moist alpine meadows, and mature forest. Five species of salmon were available beginning about July 1 until late October throughout the western half the study area.

## Materials and methods

### Field sampling

Hair samples were collected from grizzly bears using baited barbed wire hair traps. Only liquid attractant was used at bait sites and they were distributed systematically across the study area using a grid [18]. We also placed barbed wire across trails near salmon streams although they were not systematically distributed due to the heterogeneous nature of spawning habitat. We did search for spawning areas along all plausible streams in the study area. Sampling occurred from July 15 to September 17, 2004 and was designed to generate a precise population estimate.

Our field methods were approved by the Animal Care Committee and the Resource Inventory Standards Committee for the Government of the Province of British Columbia. No permit was required for this work because the methods were non-invasive and did not involve capturing an animal and, the animal of study was neither endangered nor protected by law. This study took place entirely on public land. All tissue samples from dead animals except marmots were donated by hunters or Provincial Conservation Officers and these animals were all killed and collected during a legal hunt. No animals were specifically killed for this study. Samples of 5–10 marmot hairs came from the Museums of University of Alaska-Fairbanks and University of California-Berkley from specimens legally collected by museum staff. These samples were provided because they contributed minimal damage to the specimens and these specimens continue to be held by these institutions.

The study area was split into coastal and interior areas based on the presence of salmon in each sub-drainage and, each individual bear that we detected was assigned to a life history group based on their detection locations. Coastal bears were assumed to prefer and rely on salmon for the majority of their diet while interior bears were assumed to select foods of terrestrial origin. Bears that were detected in both parts of the study area were assigned to the coastal group if any of their locations were within 100 m of a salmon spawning area where bears were known to fish. Additional sampling during 2005 resulted in many repeated detections (1–9 per individual) and all detection data were used to assign bears to a life history group. Diet data were not considered when assigning bears to a life history group and thus group assignments were putative.

### Hair preparation and analysis methods

Standard microsatellite genotyping of six loci was used to identify individuals that left hair samples [19]. Lab methods and procedures used to control genotyping errors are described in Paetkau [20] and were subsequently further tested [21]. Additionally, a larger suite of 14 micro-satellites was analyzed for each individual identified to further test for genotyping errors. Genetic analysis was conducted by Wildlife Genetics International in Nelson, BC, Canada.

Isotope analysis was conducted for all individual bears detected during hair sampling in 2004 for which entire guard hairs were available (n = 91 bears). Complete guard hairs (2 to 4 per sample) were cut in thirds based on length for 40 bears. Each portion of the complete guard hair (i.e., tip, middle and base) was analyzed separately to investigate seasonal variation in diet. Single complete hairs were analyzed for the remaining 51 bears. The longest hair available was chosen and hair length and the presence of a root were recorded. The presence of a root confirms the entire hair was analyzed assuming no portion of the tip had broken off.

The date when a hair sample is collected may influence the isotope signature observed because the sampled hair may have grown the previous year or, it may be from the year of sampling and hence may not be finished growing. Hair samples that were grown during the year of sampling will represent diet for only part of the period of hair growth. We choose hair samples that were collected as early in the sample period as possible and presumed these hairs were grown the previous season. We also selected the longest hairs in the sample for isotope analysis. We choose these samples because we wanted to measure annual diet, to the extent possible with guard hair. Guard hair begin shedding in late May or June and presumably new hair begin growing soon thereafter [15], although hair may begin growing at different times [13] complicating the estimation of diet.

Bear guard hair are thought to grow at constant rates [13,22]. If this is true then there must be considerable variation in the start time for growth because there is large variation in guard hair length in northern bears. Alternatively, different types or lengths of hair may grow at different rates [23]. Bears in northern latitudes have about 5 months to grow guard hair (June-October) and interior bears commonly have guard hair >12 cm long [13]; coastal bears appear to have shorter hair. The fastest hair growth rate for guard hair is likely to be 3 cm/month because the longest hairs observed in interior bears was about 15 cm [13] and the maximum growth period for many of these bears was five months given their long denning periods [24]. Coastal bears more commonly have guard hair 10 cm long (this study) and these hairs appear fully grown in October based on observations of live bears. This observation suggests guard hair also may grow 2 cm/month in some cases, which is more similar to the 1.5 cm/month rate observed by Felicetti et al. [22] in captive bears. Shorter growth periods imply faster growth.

We used hair growth rates to assign each sample to a year of sampling. Sample collection date was assumed to be the mid-point between the date of setting and checking the trap. We also assumed hair began growing June 1 and ended October 31. Hair that grew >3 cm/month based on the June-October growth period were considered to have grown the year previous because the growth rate would have been even higher if they had grown the year they were sampled (usually about double). Hair that grew >2 cm/month were also considered to have been grown the year previous though with less certainty. Hair that grew < 2 cm/month were considered to have grown the summer of sampling, and the end date of the growth period was thus the sample collection date not October 31. In summary, we had three classes of samples: those where we were quite sure they were grown the previous year, some we were not as certain they were grown the previous year, and those which were likely grown during the summer we sampled.

Foods for which there was no published isotope data were collected in the field and subjected to isotope analysis. We were unable to collect many marmot samples in the field and requested hair samples from museums that had specimens from near our study area. Where possible we ran hair and muscle samples for the same individuals to test whether hair generated similar results to muscle at the time of death.

All hair samples were washed for 2 hours at room temperature in 2:1 CHCl3:CH3OH, rinsed four times with ultrapure water, and air dried at room temperature for a minimum of 72 hours. If present, root bulbs were removed and returned to the sample package. Meat, skin, and plant tissue were washed four times with ultrapure water, freeze dried, and ground to a fine powder before analysis. Fat was not extracted from food or bear hair samples because it is a key macronutrient for bears and is highly selected for in fall and perhaps other seasons. Isotopic analyses of carbon and nitrogen were done at The Water Resource Sciences Lab, UBCO, Kelowna, Canada using an elemental analyzer (EA) coupled to an isotope ratio mass spectrometer. Samples were combusted and the tissue carbon and nitrogen converted to CO_2_ and N_2_, which were separated chromatographically with a Euro EA. Ratios of ^15^N/^14^N and ^13^C/^12^C in the gases were measured with a Micromass IsoPrime isotope ratio mass spectrometer with standard reference gases (CO_2_ and N_2_), and calibrated to National Institute of Standards and Technology calibration standards. Replicate standard reference materials (valine) were run at the start and end of the sample run, and after every nine samples. Standard deviations of ^15^N/^14^N and ^13^C/^12^C to standard reference materials were 0.49 ‰ and 0.02 ‰, respectively.

Sulfur analysis was conducted at the US Geological Survey lab in Boulder, USA. Sulfur samples (~1–2 mg) were weighed into tin boats (5 mm × 7 mm) and ~1.5 mg of V^2^O^5^ added prior to sealing. Sulfur isotope measurements of hair and putative diet items were analyzed by continuous flow isotope ratio mass spectrometry. The analytical set-up consisted of a Thermo GasBench II interfaced on the back end of an elemental analyzer (Costech Analytical) operated under normal conditions for sulfur analysis. The GasBench, which is coupled to a Thermo Delta Plus XP, provides a means of automated cryo-trapping of SO_2_ analyte gas for the specific objective of measuring S isotope ratios of small organic samples, similar to the design by Fritzsche and Tichomirowa [25]. Samples were scaled to V-CDT using internal laboratory sulfate standards that have been calibrated to NBS 127 (+21.1 ‰) and IAEA-SO6 (-34.05 ‰). Analytical precision specific to sulfur isotope analysis by cryo-trapping was +/- 0.4 ‰ or better.

### Diet analysis

Diet can be partitioned only if potential foods are isotopically distinct [26]. Foods with similar isotope signatures are therefore often grouped together for analysis and general food baseline isotope ratios are used to estimate diet [27,28].

We calculated source baseline isotope signatures for moose, marmot and mountain goat from samples collected from our study area, or nearby, in the case of marmots. Other mammal species were either not present, such as ground squirrels (*Spermophilus columbianus*), or rare, such as deer (*Odocoileus hemionus sitkensis*). Ants from Princess Royal Island on the mid-coast of BC had similar δ^13^C and δ^15^N signatures to moose in this study (T. Shardlow, Department of Fisheries and Oceans, Nanaimo, BC retired, pers. comm.). Harbor seals (*Phoca vitulina*) were found throughout the salmon inhabited streams in the study area during summer, but there were no observations of grizzly bears capturing seals. Any consumption of seal or other marine organisms would likely have been assigned as salmon due to similarities in isotopic values for salmon and other marine organisms. Because we only analyzed 2 of the 6 available salmon species, we used the general salmon baseline for δ^13^C and δ^15^N values presented by Mowat and Heard [12], which included isotopic data from the 5 common salmon species (Table 1). We used our data to calculate the δ^34^S baseline for salmon which included data from coho (*O*. *kisutch*) and sockeye (*O*. *nerka*) salmon only (Table 1). Bears eat many species of plants through the year hence developing a plant baseline, or baselines, based on a local collection would be difficult and complex. Instead, we used the generalized baselines presented by Mowat and Heard [12] that were calculated from individual bears that were assumed to have eaten a vegetarian diet based on their δ^15^N values (Table 1).

**Table 1:** Mean isotope ratios and generalized isotope endpoints, corrected for trophic fractionation, that we used to calculate diet proportions for grizzly bears. Data for salmon and plants for δ^13^C and δ^15^N are taken from Mowat and Heard (2006); endpoints for δ^34^S and generalized meat are derived from data from this study.

Food class	δ^13^C	Δδ^13^C	SD	δ^15^N	Δδ^15^N	SD	n^1^	δ^34^S	Δδ^34^S	SD	n
North Coast generalized meat	-24.8	-22.8	1	2.5	6.5	1	107	0.6	1.6	4.5^2^	31
Generalized anadromous salmon	-19.9	-18.9	1	12.5	15.2	1	338	19.1	15.3	0.5	21
Generalized plant baseline	-26.6	-24.6	2	-2.8	2.8	3	200	-2.0^3^	-0.4	4.2	44

^1^This sample size applies to both carbon and nitrogen.

^2^This is the mean of the SD for moose, goat and marmot.

^3^This is the mean for all bear samples where δ^15^N < 3.7.

Consumers preferentially accumulate heavy isotopes and this discrimination process must be accounted for when calculating diet fractions [29]. Felicetti et al. [15] calculated the trophic shift of δ^13^C, δ^15^N and δ^34^S from diet to blood plasma by feeding captive bears mixed diets while building on previous work by Hilderbrand et al. [11]. We used the equation from Felicetti et al. [15] to calculate trophic fractionation between diet and hair for δ^15^N because this equation was specific to our study species. The diet-plasma relationship for δ^13^C was less precise than the relationship for δ^15^N. In addition, there is evidence that diet to hair fractionation is greater in bears, and other mammals, than diet to plasma [3,30,31]. Previous authors have reduced bear hair δ^13^C values by 1–2 ‰ to account for the greater fractionation of δ^13^C in hair [3,12]. Given the uncertainty in the diet-hair relationship for δ^13^C we followed other recent authors [14,32] and used a fixed fractionation value of 3.7 (SD = 0.2). Discrimination for sulfur decreases with increasing δ^34^S in the diet [15,33]. We used the relationship presented in Florin et al. [33] to calculate sulfur fractionation because they tested a broader range of values with a larger sample size than Felicetti et al. [15]. However, the relationships presented in the two independent studies were nearly identical and very precise.

We calculated diet proportions using the R package Stable Isotope Analysis in R (SIAR; [34]). We used informative priors when calculating diet ratios for coastal and interior groups of bears based on previous results near the study area [3,12]. For the coastal analyses we used priors of salmon = 0.4, moose = 0.05, goat = 0.05, marmot = 0.05, vegetation = 0.45 and a standard deviation of 0.1; these are mean diet proportions for the group that must sum to 1. For interior analyses we used priors of salmon = 0, moose = 0.2, goat = 0.1, marmot = 0.1, vegetation = 0.6 and again a standard deviation of 0.1 (S6 File). We used uninformative priors to estimate diet for individual bears because previous work has shown that individual bears may assimilate δ^13^C and δ^15^N entirely from plant matter or almost entirely from salmon [3,12]. Similarly, an individual that finds a moose or goat carcass could have a seasonal diet that is nearly pure meat during a single season. We did not incorporate concentration dependence because we did not have all the information required to do so [14], particularly in regards to sulfur.

Sectioned hair samples were used to examine the seasonal timing of the consumption of meat. Ungulate hunting was likely most common in spring after young were born while marmot hunting was likely most common in fall after they were hibernating. We used whole hair samples from all 91 individual bears to compare the diet between putative coastal and interior groups and the sexes (S7 File). Whole hair values for sectioned samples were derived by taking the mean of the 3 sectioned values.

### Other statistical analysis

Pearson correlation was used to test for simple bivariate relationships among continuous variables. Multiple linear regression was used to investigate the relationship between bear sex, the presence of a root on a hair, hair length, and hair collection date and the stable isotope ratio.

## Results

### Precision of isotope measures

Measurement error varied among bears and samples. Multiple hairs from the same sample were analyzed for two bears; these represent different hairs taken from the same bear on the same day. One set of hairs had low variation among measures [Standard Deviation (SD) δ^13^C = 0.03, SD δ^15^N = 0.04, SD δ^34^S = 0.45, n = 3] while another had much higher variation for all three isotope ratios (SD δ^13^C = 0.50, SD δ^15^N = 0.17, SD δ^34^S = 4.27, n = 4 for δ^13^C and δ^15^N and n = 3 for δ^34^S). Different samples, which were collected at different places and times, were run for three bears and variation among samples was modest (SD δ^13^C = 0.13, SD δ^15^N = 0.12, SD δ^34^S = 1.09, n = 3). Variation in δ^34^S values was about an order of magnitude greater than δ^13^C or δ^15^N values. We conclude that process error, even between hairs within a single sample, was much greater than measurement error of the analytical instruments. Much of this error appears to be variation among hairs from the same bear and year and hence does not measure an external ecological process but rather differences in the physiological growth process.

### Isotope ratios of potential grizzly bear foods

Paired samples of marmot hair were depleted for all three isotopes compared to muscle (Table 2). Sockeye and coho skin and muscle samples had similar stable isotope ratios except coho skin was depleted for δ^13^C compared to muscle (Table 2). One sample of coho eggs was depleted for δ^13^C, enriched for δ^15^N, and similar for δ^34^S compared to coho muscle samples (Table 2). Coho eggs had higher δ^15^N and similar δ^13^C signatures compared to muscle from spawning adults in southcentral Alaska[35]. Coho from southcentral Alaska had higher δ^13^C and similar δ^15^N to data presented here [35]. There was greater variation in stable isotope signatures among species of salmon [35,36] and citations therein. The δ^34^S values for coho and sockeye salmon were similar for both species and all tissue types and, similar to the value for Chinook (*O*. *tshawytscha*) presented by Felicetti et al. [15] (δ^34^S = 19.5, sample size not given), which presumably originated from south of this study area near Seattle, USA. The sulfur signature for salmon was extremely precise compared to other foods (Table 3).

**Table 2:** The mean difference between isotope ratios of muscle and hair or skin for three potential grizzly bear foods. Delta values are the average difference compared to muscle. A single sample of coho eggs was compared to the mean coho muscle values because this sample was taken from a unique fish.

Species	Δδ^13^C	SD	Δ δ^15^N	SD	Δ δ^34^S	SD	n
Marmot muscle—hair	-3.0	0.9	-1.6	0.8	-2.4	0.5	2
Sockeye muscle—skin	0.5	1.7	0.3	0.5	0.0	0.4	6
Coho muscle—skin	-2.3	0.7	-1.0	1.3	0.6	0.5	4
Coho muscle—egg	-1.5		2.7			0.5	1

**Table 3:** A comparison of mean isotope ratios of various tissue types for four potential grizzly bear prey species from the lower Stikine valley of northeast British Columbia.

Species	δ ^13^C	SD	δ^15^N	SD	δ^34^S	SD	n
**Herbivores**							
Moose hair	-26.5	0.6	3.5	0.8	-2.7	4.4	2
Moose muscle	-27.1	0	1.3	0.9	-4	4.6	2
Marmot hair	-24.5	1.1	1.2	2.0	5.4	6.8	14
Marmot muscle	-26.2	0.6	2.7	0	-3	0.7	2
**Salmon**							
Sockeye skin	-21.3	1.2	11.3	1.9	19	0.7	6
Sockeye muscle	-20.8	1.5	11.7	1.5	19	0.5	6
Coho skin	-17.8	0.7	11	2.3	18.8	0.4	4
Coho roe	-22.8		11.5		19		1
Coho muscle	-20.1	1.1	10	2.7	19.5	0.2	4

**Fig 1:**
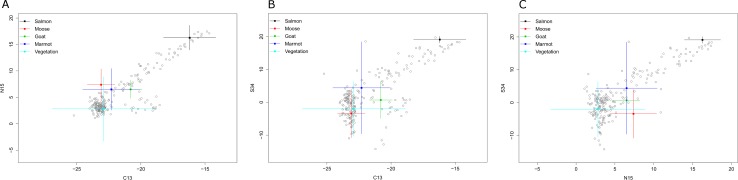
Mixing diagrams of grizzly bear hair. δ^13^C, δ^15^N, and δ^34^S values of hairs sectioned into 3 parts (n = 40 individuals and 120 measures) and whole hairs (n = 51 individuals) from samples collected during July-September, 2004 on the lower Stikine river of British Columbia, Canada.

Isotope ratios of terrestrial mammal species were similar given the variation around the means (Fig 1). Marmots had higher δ^34^S values than moose or mountain goats but variation in δ^34^S values was large (Tables 2 and 3). Moose were depleted in δ^13^C and δ^34^S but had similar values for δ^15^N compared to marmots and mountain goats. We used the values in Table 4 as endpoints for moose, marmot and mountain goats in diet analysis. We reduced the marmot hair samples by 1.5 for δ^13^C because hair was enriched for δ^13^C (Table 3) and this has been observed for other species [3]. We did not reduce the δ^13^C values for moose and goat hair because hair was not enriched for these 2 species (Table 3). This may be explained by the fact that hind-gut fermenters break their food down to constituent molecules which does not allow shunting of fat directly to build hair which is the likely cause of the enriched value for δ^13^C in single gut digesters like bears and marmots.

**Table 4:** Mean isotope ratios for seven potential grizzly bear foods from the lower Stikine valley of northeast British Columbia. δ^13^C values for marmot hair samples were reduced by 1.5. ^b^*n* = 1 for sulfur in this category.

Species	δ ^13^C	SD	δ^15^N	SD	δ^34^S	SD	n
**Herbivores**							
Moose hair and muscle	-26.8	0.5	2.4	1.4	-3.4	3.7	4
Marmot hair and muscle	-26.0	1.1	1.4	1.9	4.4	7.0	16
Mountain goat hair	-24.5	0.4	1.4	0.7	0.7	2.8	11
**Salmon**							
Sockeye muscle and skin	-21.0	1.3	11.5	1.7	19.0	0.6	12
Coho muscle, skin and roe	-18.9	1.5	10.5	2.4	19.2	0.4	8
**Plants**							
Skunk cabbage tissue	-30.3	2.7	3.5	1.5	-8.8	8.9	8
Huckleberry fruit without seeds	-30.0	0.2	0.0	0.2	3.1	^b^	2

Skunk cabbage (*Lysichiton americanus*) had unusually high δ^15^N values (Table 4; [3]) and low δ^34^S values. Most terrestrial plants have negative values for δ^15^N [12,15] and values near zero for δ^34^S [15], similar to our results for huckleberry (*Vaccinium membranaceum*; Table 4). The sulfur baseline for plants was calculated from the δ^34^S value for all grizzly bear hair samples where δ^15^N was <3.7, after Mowat and Heard [12]. This δ^15^N value was the mean value for four bear populations that were known to eat little meat plus one SD. Generalized baselines used to estimate diet are summarized in Table 1.

### Grizzly bear isotope ratios

Grizzly bear stable isotope values were correlated for all 3 isotopes we measured which was demonstrated by the roughly linear relationships in 2-dimensional space (Fig 1). Values of δ^13^C and δ^15^N are often correlated in bear samples [3,37]; the flat part of the relationship at low isotope values is likely due to the variation in δ^13^C values among plants foods [12]. One sample had high δ^15^N but low δ^13^C and δ^34^S, which can only currently be explained by near total reliance on terrestrially derived meat. Two other samples had high δ^34^S but moderate to low δ^15^N, which can only be currently explained by consumption of white-bark pine nuts or perhaps other mast [15]. All data generated for this study are available in the Supporting Information for this paper.

### Timing of sample collection and hair length

**Fig 2:**
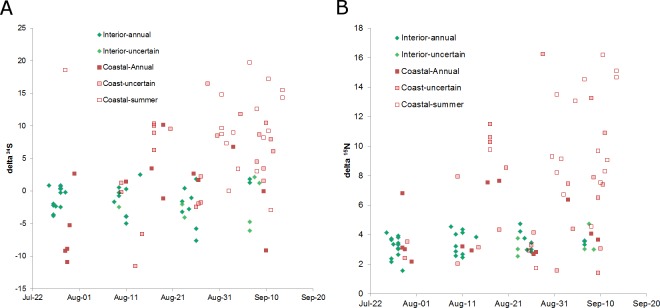
Stable isotope values vs date of sample collection. Sample collection date may be up to 14 days after samples were removed from the bear because hair traps were checked every 1–2 weeks. The longest guard hair available were selected from each sample. Some of these hairs were sectioned in thirds by length to analyze seasonal diet, the average of the 3 sectioned values is presented here. Grizzly bears detected in drainages with spawning salmon are shown in red symbols (n = 54). Green symbols indicate bears were detected in drainages without spawning salmon (n = 40). Filled symbols indicate the hair was likely from an entire season of growth, lighter fill means the period of growth is less certain though still likely from an entire season of growth and, unfilled symbols indicate the hair were likely grown the year of sampling and hence represent a partial year’s growth. Salmon began to be available to bears in mid-July but were not widespread until mid-August.

**Fig 3:**
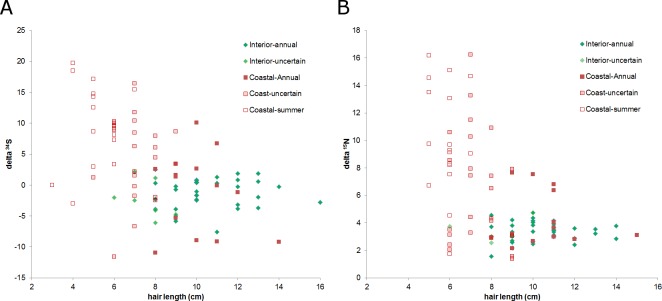
Stable isotope values vs hair length. Isotope signatures were based on analysis of the longest guard hairs available in each sample. Hair length was the average of all hairs when more than one hair was included in the isotope analysis. Some of these hairs were sectioned in thirds by length to analyze seasonal diet, the average of the 3 values is presented here. Grizzly bears detected in drainages with spawning salmon are shown in red symbols (n = 54). Green symbols indicate bears were detected in drainages without spawning salmon (n = 40). Filled symbols indicate the hair was likely from an entire season of growth, lighter fill means the period of growth is less certain though still likely from an entire season of growth and, unfilled symbols indicate the hair were likely grown the year of sampling and hence represent a partial year’s growth.

Mean date of hair collection was 14 days earlier for interior bear samples (13 August, range 26 July-9 Sept) than coastal samples (27 Aug, range 29 July-14 Sept; Fig 2). Hair selected for isotope analysis were longer from interior samples than coastal samples (interior = 10.1 cm, SD = 2.14, n = 40; coast = 7.3 cm, SD = 2.21, n = 54). Hair length declined with the date of sample collection (r = -0.43, n = 94, P < 0.001) and this was most apparent in the coastal sample group (Fig 3). The shorter hairs were likely partially grown hairs from 2004 while the longer hairs were likely from the previous year. Low δ^15^N and δ^34^S values were observed for all possible collection dates and hair lengths but, high δ^15^N and δ^34^S values were only observed later in the collection period for shorter hairs only (Figs 2 and 3).

Regression analysis suggested that interior males had δ^13^C ratios 1.9 ‰ (SE = 0.39) higher than females. Hairs with roots were enriched by 0.9 ‰ (SE = 0.36) compared to hairs that did not have roots but, neither hair length nor collection date were related to δ^13^C (F = 7.27, r^2^ = 0.49, n = 35, P < 0.001). None of the above four variables (bear sex, hair length, date of collection, presence of root) were related to δ^15^N or δ^34^S (P > 0.39 and 0.35 respectively, n = 38 bears) for interior bears. Isotope ratios were not different between sexes in the coastal group (P > 0.28, n = 39). Sulfur isotope values increased with collection date and decreased with hair length in the coastal sample (F = 7.42, r^2^ = 0.30, n = 38, P = 0.002) as did δ^13^C (F = 3.67, r^2^ = 0.09, n = 39, P = 0.06) and δ^15^N (F = 2.85, r^2^ = 0.07, n = 39, P = 0.1). In summary, for whole hairs from interior bears, the presence of a root bulb, the length of the hair, collection date and the sex of the bear were not consistently related to isotope values. For the coastal group, isotope signatures declined with hair length and increased with the date of sample collection but the presence of a hair root and the sex of the bear were not related to the values of any of three stable isotopes.

### Discerning terrestrial meat sources in the grizzly bear diet

Based on δ^13^C and δ^15^N data, the diet fractions for moose, mountain goat and marmot had lower 95% credible intervals of <1% for both coastal and interior groups of bears, while upper credible intervals varied from 19 to 34%. The mean diet fractions for the three species varied from 8–17% for both areas. There was only small improvement in the assignment of the three mammal prey when sulfur was added to the mixing model. Four lower credible intervals were still <1% and upper intervals varied from 19 to 30%. The diet fraction for interior bears was 17% moose (CI 5–30%) and 8% goats (CI 1–25%) for the coastal group using data from all three isotopes. The improvement in results with the addition of sulfur to the dataset was less when no priors were used.

At the individual level, the addition of sulfur data identified some bears that had probably consumed one of the 3 mammalian prey species. Individual analyses using δ^13^C and δ^15^N generated diet fractions with lower intervals of zero for all 3 prey species, 40 bears and 3 seasons (360 estimates in total). When sulfur was included in the diet analysis, 18 of these 360 estimates had lower credible intervals >1% (range 1–21%); 2 of these were for moose and 16 were for marmot. For comparison, credible intervals for the vegetation fraction were >1% for 89 of 120 observations using two isotopes and 88 of 120 using three isotopes. Similarly, for the salmon fraction credible intervals were >1% for 31 of 120 observations using two isotopes and 33 of 120 using three isotopes. One interior bear appeared to have eaten more mountain goat than other bears but the diet fraction assignments were very imprecise for this food source and the lower credible interval was <1%. The group analysis suggested interior bears ate mostly moose as a meat source. In conclusion, the addition of sulfur data to the diet analysis of these samples did not change the diet fractions or markedly improve the precision of the predictions when the results were presented by season and life history group. The sulfur data did identify 18 samples where the consumption of two of the three mammal prey was probable however, in 271 other cases the diet fraction was estimated to be >10% yet the precision of the prediction encompassed zero.

### Does sulfur increase the precision of assigning the marine diet portion?

The inclusion of sulfur to the diet calculation of the coastal group of whole hair samples generated nearly identical salmon diet fractions to the use of δ^13^C and δ^15^N alone, although the credible interval width was reduced by 8%. In a similar analysis, we combined terrestrial prey signatures because all three were similar and previous researcher have often chosen to do this. Again, diet fractions were very similar with and without sulfur and the reduction in credible interval width for the salmon diet fraction was trivial.

### Seasonal trends in diet

**Fig 4:**
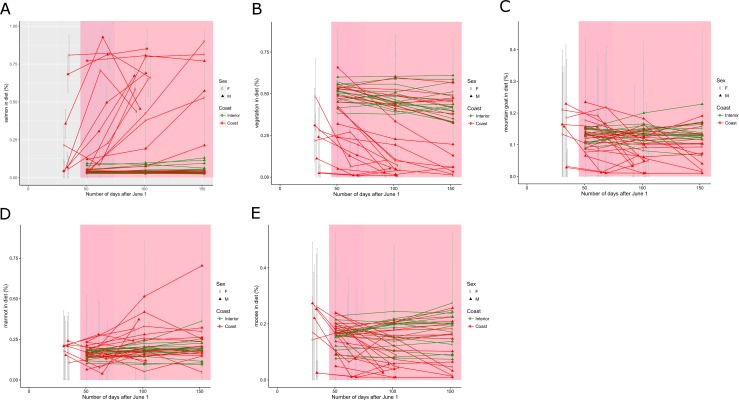
Diet of individual bears by season. Guard hairs for each sample (n = 40) were cut into 3 equal lengths. We assumed hair began growing June 1 and finished Oct 31. Each point depicts the diet between it and the previous x-value which was June 1 for the first segment of all samples. For hair samples that we believed came from the year the sample was collected, the last day of growth was the collection date. Observation date was calculated by dividing the difference between the beginning and end of hair growth into thirds. Grizzly bears detected in drainages with spawning salmon are shown in red symbols (n = 23). Green symbols indicate bears detected in drainages without spawning salmon (n = 17).

As expected, salmon consumption increased through the summer but four bears had high salmon fractions during the month of June when salmon were not known to be available (Fig 4A). These four bears were located near two streams that were known to support early chinook runs (July); spawning salmon arrived in most other streams beginning in August. No bears that were detected in the east side of the study area, which does not support spawning salmon, had meaningful salmon fractions although, some individuals that lived in the west side of the study did not have salmon fractions in their diet either. Eight of 13 coastal bear samples known to be from the previous year had salmon fractions <5% and, 7 of 25 coastal samples that may have been from the previous year had salmon fractions of <5%. Interior bears had high vegetation fractions in all 3 seasons and so did some coastal bears (Fig 4B). Coastal bears that ate salmon ate progressively less vegetation through the summer. Three coastal bears had vegetation diet fractions of zero in all seasons. Mountain goat tended to be eaten by coastal bears early in the year (Fig 4C) while marmots were consumed in the summer and fall (Fig 4D). Six coastal bears may have consumed moose early in the year while interior bears ate moose more consistently throughout the year (Fig 4E).

## Discussion

The addition of sulfur isotope data to estimate the proportional contribution of salmon or terrestrial sources of meat to the diet of grizzly bears did not greatly increase precision over the use of carbon and nitrogen isotope data alone. Measures for all three isotopes were correlated, which limited the separation of the consumers in the mixing space. And, isotope values of all three terrestrial herbivores were very similar, which further reduced separation. Foods that have isotope signatures that are much different from other foods of interest will generate more precise diet measures. All terrestrial herbivores have similar δ^13^C, δ^15^N and δ^34^S values ([12,14], this study) so the use of sulfur will not likely help separate these foods for more precisely estimating diet in other places either unless C_3_ plants are a food source for some of the herbivores and not others. Sulfur signatures for salmon are much higher than for terrestrial food and nearly fixed at 19 ‰. However, the variation in signatures of non-salmon foods, especially plants, added considerable uncertainty to the models. The use of δ^34^S to estimate population level diet will require more local data on non-salmon foods to improve markedly upon estimates using stable carbon and nitrogen isotopes alone. Plant sulfur signatures will vary locally because they take sulfur from soils, groundwater and precipitation, which often have quite different isotope ratios, and there is great variation in the amount plants fractionate sulfur [17]. The use of sulfur to estimate terrestrial diets will likely require precise mapping of local diet items. Future researchers with similar goals to this study may want to consider using other markers such as elemental proportions or ratios that are not correlated to carbon and nitrogen values and hence show better separation in the mixing space.

The use of three stable isotopes helped to identify individuals that had diets that were unusual compared to the average diet of the population. For example, several bears had enriched δ^13^C values while the δ^15^N and δ^34^S values were low and hence indicative of a plant-dominated diet. This may be the result of a selected plant being enriched for ^13^C. Similarly, several coastal bears expressed δ^15^N that would suggest the consumption of salmon but δ^34^S values were indicative of a diet dominated by terrestrial food sources. In some of these cases the δ^13^C value was similar to a plant-based diet while in others the δ^13^C value suggested the consumption of terrestrial meat (Fig 1). These bears may have consumed a large proportion of terrestrially derived meat or, foraged heavily on plants with enriched δ^15^N such as skunk cabbage. Our data suggest 15 bears ate measurable amounts of marmots in summer or fall. Many other individual bears also had sizable diet estimates for the three herbivores though the lower credible interval was zero. In fact, the three herbivore signals were so confounded that for many individuals the diet fractions for all 3 sources had lower credible intervals of zero and a total terrestrial meat fraction of 30–50%. Despite this poor precision, our data suggest that both male and female bears in the Stikine River valley hunt marmots and capture them regularly enough that they contribute measurably to their diet. Moose were consumed less frequently which is likely explained by their lower abundance compared to other food sources. In addition, guard hairs only sample diet between late spring and fall. If bears are preferentially hunting or scavenging terrestrial prey in early spring, such as moose calves or winter killed mountain goats, this will not be reflected in our diet estimates. Similarly, if bears preferentially dig for marmots in late fall before denning, this too may not be reflected in our measures of diet.

Isotope data demonstrated that many grizzly bears rely on salmon in the western portion of the study area where salmon occur. Salmon consumption begins in July and continues to the end of guard hair growth in the fall (Fig 4A). These data confirm the importance of the summer chinook and sockeye runs and the late fall coho runs to many resident bears. But, about one third of the bears that were detected in the coastal portion of the study area did not appear to consume salmon during the year of our study (Fig 4). Coastal bears are known to rely heavily on salmon for their nutrients [2,12] and this is supported by results from the adjacent area of mainland Alaska [38]. Our data suggest some bears living in salmon bearing drainages may not consume salmon, even when it is available in their home range, but continue to exploit the productive upper elevation portion of the ecosystem during the salmon season, perhaps to avoid other bears [3]. Extra-territorial movements to salmon streams by bears that live in areas that do not support salmon have not been documented for grizzly bears [18], which is supported by our data here.

Grizzly bears to the north and east of the our study area on the Edziza and Spatsizi plateaus acquired about half their nutrients from terrestrial prey, most likely ungulates [12]. Our data suggest bears in the lower Stikine river area also derive a measurable portion of their nutrition from terrestrial prey. The coastal group appeared to prey on herbivores to a similar extent compared to the interior group, in contrast to bears living on nearby coastal islands which appear to eat herbivores rarely [2,38].

Many observers have noted that coastal grizzly bears have shorter hair than interior bears. No bear that had high δ^15^N or δ^34^S values had guard hair longer than 11 cm while bears with moderate or low δ^15^N and δ^34^S values had guard hairs up to 16 cm long (Fig 2). Interestingly, several bears that we classed as coastal, because they were detected in a drainage that supported spawning salmon, had guard hair longer than 12 cm and no salmon in the diet. The mechanism for the difference in pelage is unknown but our data suggest that both phenotypes can exist in the same area and that the short-haired phenotype eats salmon while the long-haired phenotype does not, despite the availability of salmon in or near its home range.

Most sectioned hair samples showed the expected increase in salmon assimilation from the tip to the basal section of the hair. However, a number of individual bears had salmon in the diet before the known arrival date of salmon. Three bears had salmon fractions >50% of their diet from the period before salmon were known to be in the ecosystem. There are several possible explanations for this observation: 1) the hair may not have begun to grow until the salmon arrived in the ecosystem, 2) the bears found earlier runs of salmon that were unknown to field staff or by moving out of the study area, 3) these bears were digesting stored fat or protein from the previous year which was derived from salmon consumption. We cannot discount any of these explanations but we suspect the first explanation is the most likely to be true. Belant et al [39] also presented isotope data that suggested salmon consumption occurred before salmon had arrived in the Denali ecosystem in Alaska. Further, many researchers have presented data suggesting individual bears derived nearly all of their annual nutrition from salmon even though salmon do not occur in any ecosystems for the entire non-denning season and, observational data confirm that these same bears eat many other foods than salmon, especially plants in spring [2,3,12,32]. In Fig 4 we assumed that hair began growing June 1 and the results clearly suggested a number of bears were eating salmon during the month of June before salmon were available. We are quite certain there were no salmon available in June and only a few in July. If we assume guard hair began growing July 1 then the chronology of salmon arrival and their appearance in bear diet fits much better. If hair growth begins in summer not spring then most of our hair samples were likely grown the previous year. We discount explanation 2 above based on two years of intensive fieldwork and the observations of many other people on the land. We cannot discount the importance of catabolism of endogenous tissue as the reason for elevated isotope values in spring. Other researchers have shown grizzly bears to catabolize fat until midsummer [40]. However, the catabolism of endogenous fat is unlikely to be used to build structural tissue such as hair because it is more efficient to create, or route, amino acids from protein in the diet [31]. High δ^15^N and δ^34^S values in spring-grown hair are more likely generated by catabolism of endogenous proteins that were created during a period when the animal was consuming salmon.

Hair samples collected later in the summer were enriched for both nitrogen and sulfur suggesting that salmon consumption increased through the summer. Sectioned hairs also showed a trend to increasing salmon consumption later in the summer. The increasing relationship between date of collection and sulfur and nitrogen values in our data suggest that some guard hair removed in summer were grown the current year. These samples only index diet for a portion of the year. Some previous researchers may not have captured a complete season of growth if the hair samples they analyzed were collected at various times during the summer and fall. Samples collected in spring and fall are most certain to be annual samples but we caution that hairs may begin growing at different times [13]. We conclude that the period of hair growth is uncertain in wild bears and hence so is the temporal scale of diet analysis based on guard hair. We recognize that using the diet estimate for a sample to predict hair growth period is circular given both diet and growth period are unknown and argue for more controlled studies of this question.

This uncertainty may be minimized by selecting the longest hair for analysis because these hairs presumably are grown over the longest period, assuming a constant rate of growth (but see [23]). The suggestion that longer hair were grown the previous year, and therefore represent a longer period of growth is supported by the observation that δ^34^S values decreased with hair length. However, we collected many hair samples in September that were as long as the longest hair collected suggesting at least some guard hairs are finished growing by that time or, alternatively, that not all guard hairs are shed every year. Our data suggest the growth rate of guard hair in wild grizzly bears may be higher than 1.5 cm/month. We suggest using hairs that are longer than 10 cm for coastal bears and >12 cm for interior bears. These numbers should be adjusted as more hairs are measured, especially for coastal areas. Given the uncertainty around both the start and end of the growth period and, the variation in signatures among hairs from the same sample, using multiple hairs for each isotope analysis could reduce variation within individuals and generate a diet measure for a more standardized period.

If possible, only hairs with roots should be selected for analysis and this may necessitate coordination with genetic analysis so that hairs that have the roots removed for genetic analysis are identifiable to later users. Also, hair should be cut a standard distance above the bulb, perhaps 1–2 mm to ensure the full length of the shaft is available for stable isotope analysis. That said, our data suggest that the bias due to the use of hairs without roots is small.

This study and others have shown that there can be considerable variation in stable isotope signatures among hairs from the same bear [12,13]. Ben-David et al. [3] suggest running enough replicate samples from individual bears to reduce the variation among samples below the measurement errors. For studies where the objective is to estimate population level diet, effort may be best devoted to increasing the sample size of individuals to better sample the populations of interest. But, if the study objective is to compare diets of individual animals, then effort would be better devoted to repeated sampling of individuals over time and space.

**Fig 5:**
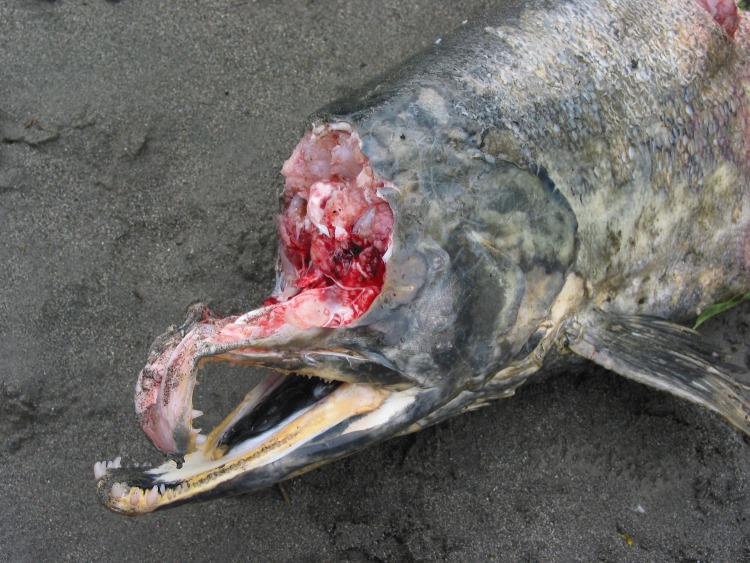
Chum salmon selective foraging. This photo shows how grizzly bears may eat only the brains of salmon when they are abundant, which was commonly observed in our study area. They also preferred the eggs of female salmon and at times choose the skin while leaving other body parts.

Our analysis of bear foods presented the first isotope data for marmot and confirmed that marmot isotope ratios are similar to other terrestrial herbivores. Salmon skin probably had lower δ^13^C values than muscle because it is higher in fat and fat is known to be depleted for δ^13^C in many organisms. This observation may be important because bears often preferentially select the brain, roe and skin when feeding on abundant salmon (Fig 5). We also found that huckleberries, a key fall food for grizzly bears in interior BC, have similar stable isotope values to other plant foods which means isotope analysis is not likely to be helpful in assessing the importance of fall berries to annual diet. Hopkins et al. [41] found sulfur was informative in separating human foods from other bear foods in Alberta, Canada. Adding sulfur as a diet marker, though not highly informative for salmon or terrestrial meat, may be quite helpful in separating plant components of the diet because there are appears to be considerable variation in plant sulfur signatures.

## Supporting information

S1 File Raw isotope data. (raw data.xls)
(XLS)

S2 File Isotope values for potential diet items for the SIAR analysis. (sources.txt)
(TXT)

S3 File Trophic correction values for the SIAR analysis. (corrections.txt)
(TXT)

S4 File Consumer (grizzly bear) data for entire guard hairs (n = 94 samples from 91 bears). (All annual samples.xls)
(CSV)

S5 File Consumer (grizzly bear) data for sectioned guard hairs (n = 120 samples from 40 bears). (All sectioned samples.xls)
(CSV)

S6 File R code for the annual analysis in Fig 1. (Stikine_annual hair_script.R)
(R) Click here for additional data file.

S7 File R code for the seasonal analysis in Fig 4. (Stikine_seasonal hair analysis_script.R)
(R) Click here for additional data file.
